# Evaluation of the effectiveness of the SurePure Turbulator ultraviolet-C irradiation equipment on inactivation of different enveloped and non-enveloped viruses inoculated in commercially collected liquid animal plasma

**DOI:** 10.1371/journal.pone.0212332

**Published:** 2019-02-21

**Authors:** Elena Blázquez, Carmen Rodríguez, Jesús Ródenas, Núria Navarro, Cristina Riquelme, Rosa Rosell, Joy Campbell, Joe Crenshaw, Joaquim Segalés, Joan Pujols, Javier Polo

**Affiliations:** 1 APC EUROPE, S.L.U. Pol. Ind. El Congost, Granollers, Spain; 2 IRTA, Centre de Recerca en Sanitat Animal (CReSA, IRTA-UAB), Campus de la Universitat Autònoma de Barcelona, Bellaterra, Barcelona, Spain; 3 Departament d’Agricultura, Ramaderia, Pesca i Alimentació (DARP) Generalitat de Catalunya, Campus de la Universitat Autònoma de Barcelona, Bellaterra, Barcelona, Spain; 4 APC Inc, Ankeny, Iowa, United States of America; 5 Departament de Sanitat i Anatomia Animals, Universitat Autònoma de Barcelona (UAB), Bellaterra, Barcelona, Spain; 6 UAB, Centre de Recerca en Sanitat Animal (CReSA, IRTA-UAB), Campus de la Universitat Autònoma de Barcelona, Bellaterra, Barcelona, Spain; Michigan Technological University, UNITED STATES

## Abstract

The objective of this study was to evaluate the effectiveness of the SurePure Turbulator ultraviolet-C (UV-C, 254 nm wavelength) irradiation equipment on inactivation of different enveloped and non-enveloped viruses in commercially collected liquid animal plasma. Specifically, *Pseudorabies virus* (PRV), *Porcine reproductive and respiratory syndrome virus* (PRRSV), *Porcine epidemic diarrhea virus* (PEDV), *Bovine viral diarrhea virus* (BVDV), *Classical swine fever virus* (CSFV), *Swine influenza virus* (SIV) as enveloped viruses and *Porcine parvovirus* (PPV), *Swine vesicular disease virus* (SVDV), *Porcine circovirus type 2* (PCV-2) and *Senecavirus A* (SVA) as non-enveloped viruses, were inoculated in bovine or porcine plasma and subjected to different UV-C irradiation doses (0, 750, 1500, 3000, 6000 and 9000 J/L) using an UV-C device developed for opaque liquid working under turbulent flow. The enveloped viruses tested were inactivated at < 3000 J/L of UV-C, being the dose needed to inactivate 4 log TCID_50_ (4D) of 1612 J/L for PRV,1004 J/L for PRRSV, 1953 J/L for PEDV, 1639 J/L for SIV, 1641 J/L for CSFV and 1943 J/L for BVDV. The non-enveloped viruses tended to have higher 4D values: 2161 J/L for PPV, 3223 J/L for SVA and 3708 J/L for SVDV. Because the initial viral concentration was <4.0 Log for PCV-2, it was not possible to calculate the 4D value for this virus. In conclusion, these results demonstrated that the SurePure Turbulator UV-C treatment system is capable of inactivating significant levels of swine viruses inoculated in commercially collected porcine or bovine plasma. It was concluded that irradiation with UV-C can provide an additional redundant biosafety feature in the manufacturing process of spray-dried animal plasma.

## Introduction

Spray-dried animal plasma (SDAP) is a protein source widely used in pig feed due to its functional components able to improve post-weaning performance and survival [1,2]. The manufacturing process of SDAP involves the collection of blood from healthy animals at a commercial abattoir that have been inspected by veterinary officials and passed as fit for slaughter for human consumption. Immediately after collection, the blood is treated with an anticoagulant, chilled, transported to the manufacturing plant and centrifuged to obtain plasma and blood cell fractions. Alternatively, the blood can be centrifuged at the abattoir and the plasma fraction is chilled and transported to the manufacturing plant. At the manufacturing plant, the plasma is concentrated by membrane filtration and spray-dried, achieving 80°C throughout its substance [2]. The process for the production of SDAP has been validated to effectively inactivate many viral and bacterial pathogens [3–14]. These data demonstrate that commercially produced SDAP is a safe feed ingredient. However, it is appropriate to evaluate new technology that could be included into the manufacturing process representing added redundant microbial inactivation steps. Typical plasma manufacturing plants are capable of processing between 25,000 mt to 450,000 mt of liquid plasma annually.

Many different techniques have been used to inactivate or remove viruses from human plasma products, i.e. intravenous immunoglobulin (IVIG). For example, solvent/detergent mixtures have been effectively used to inactivate lipid enveloped viruses [15]. However, this process requires significant volume of solvent/detergent mixtures that must be removed and disposed. Enveloped viruses can be inactivated by the addition of unsaturated fatty acids [16], or caprylic acid in combination with low pH and temperature [17,18]. While many of these methods are very effective for plasma fractions (purified immunoglobulins or albumin) these methods are not appropriate for the production of SDAP because these processes result in many denatured plasma proteins. As a rule, non-enveloped viruses are more resistant to inactivation than enveloped viruses [19]. An alternative to inactivation is removal of the virus from plasma fractions by nanofiltration [20]. However, this is not a practical option for industrial production of SDAP since the volume of product to be processed is very large and most proteins are retained in the retentate. Similarly, other removal procedures used in the human plasma industry, like precipitation or chromatography are impractical due to the large volumes used by SDAP manufacturing industry.

Ultraviolet-C (UV-C) radiation represents an alternative to chemical inactivation methods [21]. Such methodology uses a shortwave electromagnetic radiation with a wavelength of 254 nm (range of 250 and 270 nm), which induces damage in the nucleic acids. UV-C radiation disrupts DNA and RNA [22], and is therefore effective at inactivating a wide range of microorganisms. In fact, it has been extensively used for disinfection of water, surfaces and food products [23,24]. The inability of UV-C radiation to penetrate opaque liquids can be overcome by introducing adequate turbulence insuring that all of the liquid is exposed to the surface of the UV-C light [25–30]. UV-C treatment has been shown to reduce bacterial contamination while not inactivating protein functionality or growth enhancing properties of SDAP [31]. SurePure has designed a UV-C treatment system that is able to reduce the pathogen burden in milk, fruit juices, etc. [26,28,29].

The objective of this study was to assess inactivation efficiency of the SurePure Turbulator UV-C irradiation system with selected swine enveloped viruses *Pseudorabies virus* (PRV), *Porcine reproductive and respiratory syndrome virus* (PRRSV), *Porcine epidemic diarrhea virus* (PEDV), *Bovine viral diarrhea virus* (BVDV), *Swine influenza A virus* (SIV) and *Classical swine fever virus* (CSFV) and non-enveloped viruses *Porcine parvovirus* (PPV), *Swine vesicular disease virus* (SVDV), *Porcine circovirus 2* (PCV-2) and *Senecavirus A* (SVA) inoculated in liquid bovine or porcine plasma. These viruses were selected because of their economic impact in livestock production and as models of viruses from different families with various genome type and size.

## Material and methods

### Viral strains and culture conditions

#### Pseudorabies virus

*Pseudorabies virus* strain NIA3 [32], kindly supplied by Joan Plana (Fort Dodge Veterinaria, Vall de Bianya, Spain), was propagated in the PK-15 cell line (provided by the Institute of Virology (UE and OIE Reference Laboratory for CSFV, Hannover), using a standard growth media (SGM) containing minimum essential medium eagle (MEM-E; ThermoFisher, Waltham, MA, USA) supplemented with 1% penicillin 10,000 U/mL and streptomycin 10 mg/mL (ThermoFisher), 0.5% Nystatin 10,000 IU/mL (Sigma-Aldrich), 1% L-glutamine 200 mM (ThermoFisher) and 5% of heat inactivated fetal bovine serum tested free for virus and antibodies against pestiviruses (FBS; BioWest, Miami, FL, USA). PK-15 cells were grown in 175-cm^2^ flasks (Corning, Corning, NY, USA), and when cells were confluent, the medium was discarded and adsorption was performed at a MOI 0.01. Virus stock was produced in the same cells to obtain 84 mL with a viral titer of 10^8.95^ TCID_50_/mL that was used to inoculate 24 L of bovine plasma, achieving a final viral titer of 10^6.5^ TCID_50_/mL.

#### Porcine reproductive and respiratory syndrome virus

*Porcine reproductive and respiratory syndrome virus* VP21 strain [33] was propagated in the Marc-145 cell line (ATCC CLR12231) grown in SGM with 5% FBS. Cells were cultured in 75-cm^2^ flasks. When cells were confluent, the media was discarded and the adsorption was done using the virus at a MOI 0.01. After 1.5 hours at 37°C, inoculum was removed and 30 mL of medium were added. This procedure was repeated until achieving 1,800 mL of viral suspension with a titer of 10^5.57^ TCID_50_/mL that was used to inoculate 24 L of bovine plasma achieving a final viral titer of 10^4.42^ TCID_50_/mL.

#### Porcine epidemic diarrhea virus

*Porcine epidemic diarrhea virus* CV777 strain [34], kindly provided by Dr. Hans Nauwynck (University of Ghent, Belgium), was propagated in VERO cells (ATCC CCL-81) grown in SGM with 10% FBS. Cells were cultured in 175-cm^2^ flasks and when they were confluent, the media was removed and cells were rinsed twice with PBS. Finally, inoculum was added at MOI 0.001 and adsorption was done for 1 hour at 37°C. Subsequently, the inoculum was discarded, flasks were rinsed twice with PBS and MEM-E was supplemented with 1% penicillin 10,000 U/mL and streptomycin 10 mg/ml (ThermoFisher), 0.5% Nystatin (10,000 U/mL), 1% L-glutamine (200 mM), 0.05% trypsin and 0.3% tryptose. The viral stock was produced in the same cells to obtain 2,110 mL of suspension with a viral titer of 10^5.42^ TCID_50_ /mL that was used to inoculate 24 L of bovine plasma achieving a final viral titer of 10^4.7^ TCID_50_ /mL.

#### Bovine viral diarrhea virus

*Bovine viral diarrhea virus* NADL was provided by the Institute of Virology (UE and OIE Reference Laboratory for CSFV, Hannover) and was propagated in MDBK cells (provided by the Institute of Virology (UE and OIE Reference Laboratory for CSFV, Hannover), grown in SGM with 5% FBS tested free of pestivirus antibodies. Cells were cultured in 175-cm^2^ flasks. Media was discarded and the adsorption was done on confluent cells using BVDV strain at a MOI 0.01. After 1.5 hours at 37°C, inoculum was removed and 50 mL of medium were added. This procedure was repeated until getting 255.3 mL of a suspension with a titer of 10^7.87^ TCID_50_/mL that was used to inoculate 24 L of porcine plasma achieving a final viral titer of 10^4.5^ TCID_50_/mL.

#### Classical swine fever virus

*Classical swine fever virus* strain Alfort 187, provided by the Institute of Virology (UE and OIE Reference Laboratory for CSFV, Hannover), was propagated in the PK-15 cell line (provided by the same Institute of Virology, Hannover), grown in SGM supplemented with 5% of FBS free from pestivirus antibodies. A total of 160 mL of virus stock solution was produced in the same cells at a MOI 2, for a virus titer of 10^7.36^ TCID_50_ /mL that was used to inoculate 24 L of bovine plasma achieving a final viral titer of 10^5.19^ TCID_50_ /mL.

#### Swine influenza virus

*Swine influenza virus* strain H1N1 A/Swine/Spain/SF11131/2017 [35] was propagated in MDCK cell line (ATCC CCL-34) grown in DMEM (ThermoFisher, Waltham, MA, USA) supplemented with 1% penicillin (10,000 U/mL), 1% streptomycin (10mg/mL; ThermoFisher), 0.5% Nystatin (10,000 U/mL; Sigma-Aldrich), 1% L-glutamine 200mM; ThermoFisher) and 5% FBS. Cells were cultured in 175-cm^2^ flasks. When cells were confluent, the media was discarded and the adsorption was done at MOI 0.1. After 1 hour at 37°C, inoculum was removed and 30 mL of medium were added. With this procedure, 300 mL of viral suspension with a final virus titer of 10^7.2^ TCID_50_ /mL that was used to inoculate 24 L of bovine plasma achieving a final viral titer of 10^5.3^ TCID_50_/mL.

#### Senecavirus A

*Senecavirus A* isolate BRA/UEL-PR/15 was kindly provided by Dr. Amauri Alfieri (Universidade Estadual de Londrina, Londrina, Brazil) and was propagated in PK-15 cell line (provided by the Institute of Virology (UE and OIE Reference Laboratory for CSFV, Hannover), grown in SGM with 10% FBS. Viral infection was done in confluent 175 cm^2^ flasks at a MOI 0.001. After 1 hour of absorption, 50 mL of SGM were added. CPE was visible at 72 h and the flasks were frozen. Following this procedure 1,100 mL of SVA with a titer of 10^6.76^ TCID_50/_mL was produced, that was used to inoculate 24 L of bovine plasma achieving a final viral titer of 10^5.43^ TCID_50/_mL.

#### Swine vesicular disease virus

*Swine vesicular disease virus*, strain UK-72, was provided by David K.J. Mackay (European Community Reference Laboratory for Foot and Mouth Disease, Institute for Animal Health. Pirbright, UK) to Laboratorio de Sanidad Animal (Barcelona). SVDV was propagated in SK-RST cell line (ATCC CRL-2842), grown in SGM supplemented with 5% FBS. A virus stock was produced using the same cell line at MOI 0.001 to obtain 24 mL of a virus stock solution with a titer of 10^7.38^ TCID_50_ /mL that was used to inoculate 24 L of bovine plasma achieving a final viral titer of 10^6.00^ TCID_50_ /mL.

#### Porcine circovirus 2

*Porcine circovirus 2* genotype b isolate Sp-10-7-54-13 [36] was cultured in the PK-15 cell line (provided by the Institute of Virology (UE and OIE Reference Laboratory for CSFV, Hannover), grown in SGM with 10% FBS. A mix of 6 mL of virus stock and 7 x 10^6^ PK-15 cells resuspended in 50 mL of MEM-E (MOI 0.1) were added in 175 and 25 cm^2^ flasks. At 24 hours cells were treated with glucosamine to facilitate the virus infection. Forty-eight hours later, viral infection was checked by IPMA [37] in the 25 cm^2^ flask. If more than 25 positive cells were counted in a microscope field, the 175 cm^2^ flask was trypsinized and the cells were transferred to 3 new 175 cm^2^ flasks. The process was repeated until 2,316 mL of virus stock with a titer of 10^4.79^ TCID_50_ /mL that was used to inoculate 24 L of bovine plasma achieving a final viral titer of 10^3.76^ TCID_50%_ /mL.

#### Porcine parvovirus

*Porcine parvovirus* strain NADL-2 was kindly provided by Dr Albert Bosch (Department of Genetics, Microbiology and Statistics School of Biology, University of Barcelona, Spain). It was propagated in SK-RST cells (ATCC CRL-2842), grown in SGM supplemented with 5% FBS. One mL of virus stock and 9 mL of MEM-E supplemented with 1% pyruvate (Merck KGaA, Darmstadt, Germany) were added to a conical tube with 16 x 10^6^ SK-6 cells and shaken for 30 minutes at 104 rpm and 37°C. After that time, the contents of the tube were transferred to a 175 cm^2^ flask, in which 40 mL of MEM-E supplemented with 1% pyruvate were added. This procedure was repeated until obtaining 811 mL of viral suspension with a titer of 10^6.91^ TCID_50%_ /mL that was used to inoculate 24 L of bovine plasma achieving a final viral titer of 10^5.44^ TCID_50_ /mL.

Genomic data and virion size of each virus used in this study are displayed in Table 1.

**Table 1:** Virion size and genome characteristics of the viruses used in the study.

VIRUS	FAMILY	ENVELOPE	GENOME	SIZE (nm)	GENOME SIZE (Kb)
PRV	*Herpesviridae*	Yes	dsDNA	150–180	143.46
PRRSV	*Arteriviridae*	Yes	(+)ssRNA	50–65	15.43
PEDV	*Coronaviridae*	Yes	(+)ssRNA	95–190	28.03
BVDV	*Flaviviridae*	Yes	(+)ssRNA	25–120	12.57
CSFV	*Flaviviridae*	Yes	(+)ssRNA	25–120	12.3
SIV	*Orthomyxoviridae*	Yes	(-)ssRNA	80–120	13.15
SVA	*Picornaviridae*	No	(+)ssRNA	30	7.31
PPV	*Parvoviridae*	No	ssDNA	18–26	5.07
PCV2	*Circoviridae*	No	ssDNA	17	1.77
SVDV	*Picornaviridae*	No	(+)ssRNA	22–30	7.39

### Plasma

Bovine and porcine blood was obtained from EU inspected slaughter facilities from animals inspected and approved for slaughter for human consumption. Blood was collected in stainless steel containers with sodium phosphate as anticoagulant. Blood was refrigerated and transported to the APC Europe laboratory (APC-Europe S.L.U., Granollers, Spain) and plasma was separated by centrifugation. Plasma was frozen at -20°C.

Prior to virus inoculation and UV-C irradiation, plasma was thawed and filtered to eliminate potential cryoprecipitate. Before virus inoculation, a 100-mL sample of plasma was stored at –80°C to determine absence of the test virus and absence of neutralizing antibodies to the test virus.

For all viruses tested, after virus inoculation to plasma, the 24 L mixture was divided into three equal 8 L aliquots. Each aliquot was subjected to UV-C irradiation to provide triplicate analytical results.

### Virus inoculation in plasma procedure

Because antibodies against porcine viruses are not expected to be found in bovine blood, bovine plasma was inoculated with PRV, PRRSV, PEDV, CSFV, SIV, SVA, SVDV, PPV and PCV-2. Subsequent test confirmed the bovine plasma was negative for specific antibodies against the test virus. Similarly, porcine plasma was tested for BVDV antibodies by neutralizing peroxidase monolayer assay.

### Settings of UV-C system

The UV-C reactor system SurePure Turbulator used for the study was designed for opaque liquids, manufactured by SurePure Operation AG (Zug, Switzerland) and is described elsewhere [30]. Briefly, the system consists of a reactor with a stainless-steel inlet and outlet chamber with a stainless steel corrugated spiral tube between the chambers. Inside the spiral tube is an UV-C 254 nm wavelength germicidal lamp of 100-Watt (W) output (30 W UV-C output) protected by a quartz sleeve. The liquid flows between the corrugated spiral tube and the quartz sleeve. The tangential inlet of the reactor creates a high velocity and turbulence in the inlet chamber and brings the liquid close to contact with the surface of the UV-C light source. The liquid is pumped at a minimum flow rate of 4000 L/h with a Reynolds value in excess of 7500 (Reynolds number > 2,600 indicates a turbulent flow).

The UV-C dosage is expressed as J/L. The operation time of the UV-C treatment is based on the quantity of product to be treated and the flow rate of the product feed. At a flow rate of 4000 L/h, 9 s are required for 10 L of product to pass through the reactor once; thus, one turn of the product through the system is equivalent to a UV-C dose of 22.95 J/L. The UV dosage per L of liquid treated for one reactor with continuous flow was calculated as follows: Dosage = Total UV-C output per unit (W) / Flow rate (L/s) = 25.50 W / 1.11 L/s = (25.50 J/s)/ (1.11 L /s) = 22.95 J/L. Using an ammeter, the input current to the machine and its consumption were checked and it was established that they were appropriate according to the technical specifications and the data sheet of the UV-C lamp provided by SurePure.

A standard ‘Cleaning in Place’ (CIP) process as described by Keyser et al. [38] based on a treatment with NaOH 5%, was implemented prior to and following each UV-C treatment.

### Sampling

Plasma flow was stabilized at 4000 L/h with the UV lamp switched off. After 5 minutes of stable flow, a positive control (0 J/L) sample was collected into sterile container. Then, the UV-C lamp was switched on and irradiation started. 175 mL of treated plasma were collected into sterile containers at different UV-C doses (750, 1500, 3000, 6000, and 9000 J/L).

### Analysis of viral titration

Infectivity of samples was determined in target cell cultures using the microtiter assay procedure [39]. The microtiter assay was done in a 96-well plate for all tested viruses, except for PEDV. Titration of virus was done using the whole plate for every dilution, from -1 to -5 dilutions, to amplify the detection capability of the test. Final titer was expressed as log TCID_50_/mL. Once all samples were titrated and the first negative dose was found, further steps were developed to ensure full inactivation of the samples. First, 50 mL of this negative dose sample were inoculated into 10 175-cm^2^ culture flasks and incubated at 37°C for 5 days. The flasks were frozen and thawed three times. The liquid was then centrifuged at 3000 rpm and 25 mL were inoculated into 5 175-cm^2^ flasks. The flasks were incubated 5 days at 37°C. The procedure was repeated again and, finally, only one flask was inoculated with 5 mL and incubated at 37°C for 5 days. If the final flask was negative, it was considered that no viral particles were present in the initial sample; if the final flask was found positive, a titration of the plasma was repeated again on ten 96-well plates to analyze a total volume of 50 mL.

Due to the cytotoxic effect of the plasma, strong washes to eliminate serum used to propagate the cell culture and trypsin addition, PEDV was titrated by means of PFU/mL. A 12-well culture plate with 100% confluency was inoculated with diluted plasma in MEM supplemented with 0.05% trypsin and 0.3% tryptose, but without FBS. Whole plates were used for each dilution, from -1 to -5, to amplify the detection capability of the test. Conversion to TCID_50_/mL was done multiplying PFU x 0.7 (https://www.lgcstandardsatcc.org/support/faqs/48802/Converting+TCID50+to+plaque+forming+units+PFU-124.aspx?geo_country=es). Negative samples were inoculated to 175-cm^2^ culture bottles to analyze a total volume of 50 mL (10 mL by bottle), and passaged three times before being discarded as negative. If a sample was found positive, a titration of pure plasma was repeated on 10 12-well plates to analyze the same total volume of 50 mL. This procedure was intended to increase 10 times the initial analyzed volume.

### Modeling of inactivation

Microbial inactivation due to thermal and non-thermal processes can be represented by eight possible curves [40]. The GInaFiT software was used to test linear and non-linear survival curves [40], using the biphasic [41],Weibull [42], Weibull plus tail [43] and biphasic plus shoulder [40] models. The GInaFiT software has been used to test survival kinetics of different bacteria and viruses when submitted to heat treatment or UV-C irradiation [44].

The biphasic model [40,41] uses the Eq (1):

$$log10(N)=log10(N0)+log10\;(f*e^{{-k}_{max1}t}\;+(1-f)*e^{{-k}_{max2}t})$$ (1)

Where N0 is the initial bacterial concentration; t is time; f is the fraction of the initial population in a major subpopulation, (1-f) is the fraction of the initial population in a minor subpopulation, and k_max1_ and k_max2_ are the specific inactivation rates of the two populations, respectively.

The Weibull model [42] shows the Eq (2):

$$log10(N)=log10(N(0))-(\frac{t}{\delta \;}{)\;}^{p}$$ (2)

Where δ is a scale parameter denoted as the time for the first decimal reduction, and p is the shape parameter that describes concavity or convexity of the curve. If p>1 the curve shows convexity and if p<1 the curve is concave.

The Weibull plus tail model [43] uses the Eq (3):

$$log10(N)=log10\;\left(\left(10^{\text{log}N0}-10^{logN_{res}}\right)\right)*10^{(-\frac{t}{\delta }\;){\;}^{p}}+10^{logN_{res}}$$ (3)

Where *N*_*res*_ is the number of resistant microorganism subpopulation.

The Biphasic plus shoulder model [40] follows the Eq (4):

$$\text{log}\;10\left(N\right)=\text{log}\;10^{\begin{array}{c} \left(\text{N}0\right)+\text{log}\;10\;\left(\text{f}*\text{exp}\left(-\text{kmax}\;1*\text{t}\right)*\frac{\text{exp}\left(\text{kmax}1*\text{Sl}\right)}{1+\left(\text{exp}\left(\text{kmax}1*\text{Sl}\right)-1*\text{exp}\left(-\text{kmax}1*\text{t}\right)\right)+1\left(1-\text{f}\right)*\text{exp}\left(-\text{kmax}2*\text{t}\right)*\frac{\text{exp}\left(\text{kmax}1*\text{Sl}\right)}{{\left(1+\left(\text{exp}\left(\text{kmax}\;1*\text{Sl}\right)-1\right)*\text{exp}\left(-\text{kmax}\;1*\text{t}\right)\right)}^{\frac{\text{kmax}2}{\text{kmax}1}}}}\right)\\ \; \end{array}}$$ (4)

Where N0 is the initial bacterial concentration; t is time; k_max1_ and k_max2_ are the specific inactivation rates of the two populations and S1 are the degrees of freedom used for the parameter estimation by GInaFiT

The Log linear model [40] presents the Eq (5):

$$log10(N)=log10(N(0))-(\frac{Kmax*t}{Ln(10)})$$ (5)

Where N represents de microbial cell density, N0 the initial microbial cell density, t is time; k_max_ is the first order inactivation constant and Ln(10) represents de decimal reduction time.

The log-linear plus tail model [40,45] uses the Eq (6):

$${logN=\text{log}\left(\left(10^{\text{log}\;N0}-10^{logN_{res}}\right)\right)-e^{\;(k_{max}\;d)}+10^{logN_{res}}}^{\;}$$ (6)

Where *k*_*max*_ is the inactivation rate of the log linear part of the curve and *N*_*res*_ is the number of resistant microorganism subpopulation.

The log-linear plus Shoulder model [40,45] shows the Eq (7):

$$log10(N)⩵\;log10\begin{array}{c} \left(10^{\text{log}10(\text{N}(0))}-10^{\text{log}10(\text{Nres}))}*e^{\text{Kmax}*t}*\text{exp}(-\text{Kmax}\;1*t)*(\frac{e^{\text{Kmax}1*\text{Sl}}}{1+(e^{\text{Kmax}1*\text{Sl}}-1)*e^{\text{Kmax}1*t}}\right) \end{array}+10^{log10(Nres)}$$ (7)

Where N0 is the initial bacterial concentration; t is time; k_max_ is the inactivation rate and S1 are the degrees of freedom.

Using these equations, the software automatically calculates the 4D value (the dose needed to inactivate 4 log of viral load). In the case of BVDV, since the titer expressed as Log TCID_50_/mL did not achieve 4 log, the 4D value was calculated for Log TCID_50_ in 10 mL of tested sample. In the case of PCV2, the 4D value could not be calculated due to the process did not achieve 4 log of inactivation.

### Statistical analyses

Data were expressed as the mean as Log10 TCID_50_ with standard deviations of three independent replicates. Mean, standard deviations, ANOVA and F-Test for comparisons were calculated with Excel 2007 (Microsoft Office). The Tukey test was done to determine significant differences between UV-C radiation doses. Mean square error (MSE), goodness of fit in terms of root mean square error (RMSE), correlation coefficient (R^2^) and adjusted correlation coefficient (adj-R^2^) values were calculated with the GInaFiT software [40]. The inactivation model with the best fit corresponded to the model with the smallest RMSE [40].

## Results

Bovine plasma used in the studies was negative for viral contamination or neutralizing antibodies against tested viruses (PRV, PRRSV, PEDV, CSFV, SIV, SVA, SVDV, PPV and PCV-2). In addition, porcine plasma was free from BVDV and BVDV antibodies.

**Fig 1:**
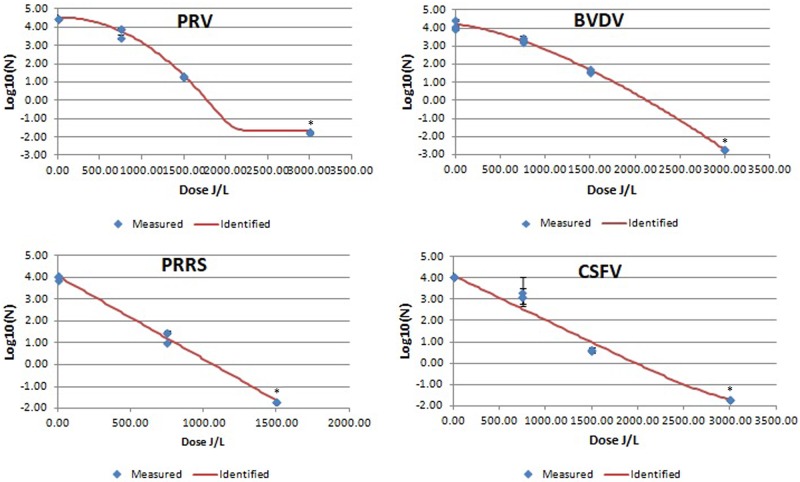
Mean PRV, PRSSV, CSFV and BVDV log 10/mL values after UV-C irradiation of bovine plasma at different UV irradiation doses. Blue diamonds indicated measured results of the viral titer at different UV-C irradiation doses expressed as mean log 10/mL (n = 3 replicates). Red line is the identified inactivation curve model. *: indicates a value below the detection limit.

**Fig 2:**
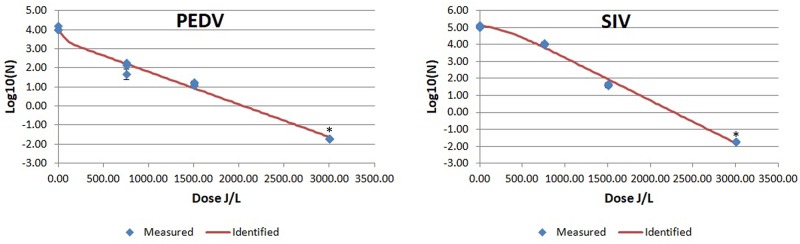
Mean PEDV and SIV log 10/mL values after UV-C irradiation of bovine plasma at different UV irradiation doses. Blue diamonds indicated measured results of the viral titer at different UV-C irradiation doses expressed as mean log 10/mL (n = 3 replicates). Red line is the identified inactivation curve model. *: indicates a value below the detection limit.

**Fig 3:**
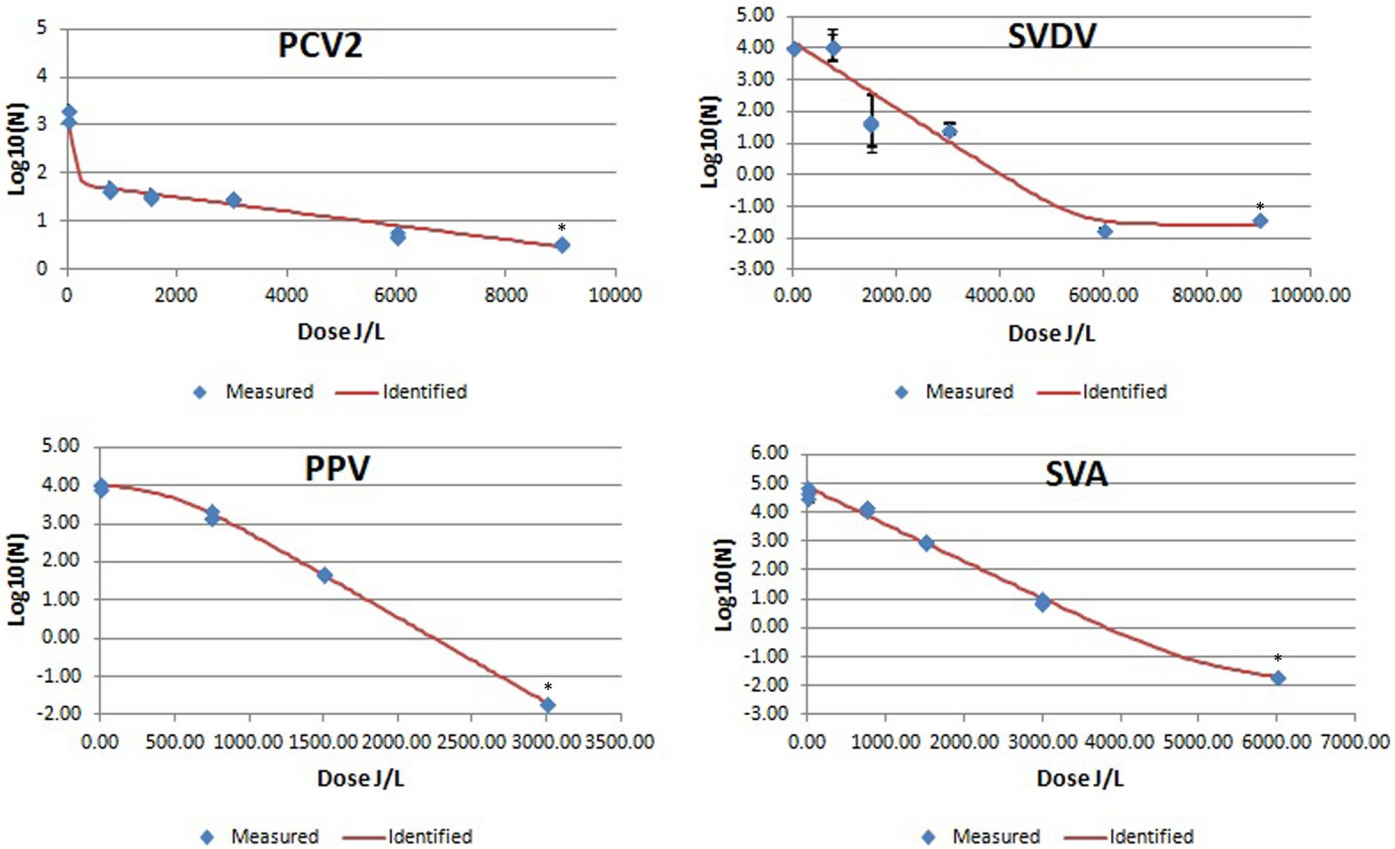
Mean PCV-2, SVDV, PPV, and SVA log 10/mL values after UV-C irradiation of bovine plasma at different UV irradiation doses. Blue diamonds indicated measured results of the viral titer at different UV-C irradiation doses expressed as mean log 10/mL (n = 3 replicates). Red line is the identified inactivation curve model. *: indicates a value below the detection limit.

The results of UV-C viral inactivation are summarized in Table 2 and in Figs 1, 2 and 3.

**Table 2:** Log reduction of viral titers expressed as Log 10 TCID_50_ at different UV-C doses and statistical parameters of models for inactivation of enveloped or non-enveloped viruses.

**Parameter**	***PRV***	***PRRSV***	***PEDV***	***BVDV***†	***SIV***	***CSFV***	***SVDV***	***PCV-2***	***PPV***	***SVA***
**Theoretical viral titer in 24 L**	6.5	4.42	4.7	4.5	5.3	5.19	6	3.76	5.44	5.43
**Viral titer (VT) before UV-C irradiation**	4.53±0	4±0.1	4.07±0.11	4.16±0.24	5.09±0.05	4.09±0.02	4.08±0	3.26±0.12	4±0.06	4.68±0.18
**VT at 750 J/L**	3.78±0.49	1.33±0.08	2.02±0.42	3.36±0.13	4.06±0.05	3.23±0.13	4.08±0.04	1.68±0.04	3.23±0.12	4.12±0.06
**VT at 1500 J/L**	1.36±0.07	BDL	1.19±0.35	1.63±0.08	1.65±0.06	0.64±0.03	1.69±0.04	1.54±0.05	1.69±0.04	2.99±0.06
**VT at 3000 J/L**	0.02±0.01	BDL	BDL	BDL	BDL	BDL	1.46±0.01	1.48±0.01	BDL	0.94±0.1
**VT at 6000 J/L**	BDL^1^	BDL	BDL	BDL	BDL	BDL	0.02±0.01	0.73±0.04	BDL	BDL
**VT at 9000 J/L**	BDL	BDL	BDL	BDL	BDL	BDL	0.04±0.02	0.55±0.01	BDL	BDL
**Best Fit model**	**Weibull + tail**	**Log linear**	**Biphasic**	**Weibull**	**Biphasic + shoulder**	**Biphasic + shoulder**	**Log linear + tail**	**Biphasic**	**Log linear + shoulder**	**Biphasic + shoulder**
**MSE**^1^	0.0195	0.0238	0.0662	0.0193	0.0023	0.0049	0.3195	0.0144	0.0054	0.0099
**RMSE**^2^	**0.1398**	**0.1543**	**0.2574**	**0.1389**	**0.0478**	**0.0703**	**0.5653**	**0.1199**	**0.0733**	**0.0993**
**R-Square**	0.9978	0.9966	0.9898	0.9979	0.9998	0.9994	0.9499	0.9855	0.9992	0.9988
**R-Square adjusted**	0.9970	0.9961	0.9859	0.9975	0.9997	0.9991	0.9432	0.9824	0.9990	0.9983
**4D reduction (J/L)**	1612	1004	1953	1943	1639	1641	3708	NC	2161	3223
**Second Best Fit model**	**Biphasic + shoulder**	**Weibull**	**Weibull**	**Log linear + shoulder**	**Log linear + shoulder**	**Weibull**	**Weibull + tail**	**Weibull**	**Weibull**	**Biphasic**
**MSE**^2^	0.0223	0.0272	0.0834	0.0389	0.0380	0.1955	0.3423	0.0233	0.0076	0.0395
**RMSE**^3^	**0.1494**	**0.1650**	**0.2888**	**0.1973**	**0.1948**	**0.4421**	**0.5850**	**0.1528**	**0.0873**	**0.1988**
**R-Square**	0.9978	0.9967	0.9855	0.9959	0.9958	0.9717	0.9499	0.9748	0.9988	0.9947
**R-Square adjusted**	0.9965	0.9955	0.9823	0.9949	0.9949	0.9654	0.9392	0.9714	0.9985	0.9932
**4D reduction (J/L)**	1786	1004	1953	1861	1723	1872	3708	NC^4^	2190	3050

^1^BDL: Below Detection Limit, being the limit of detection for each batch 1/50 mL = 0.02 viral particles/mL

^2^MSE: Mean Sum of Squared Error.

^3^ RMSE: Root Mean Sum of Squared Error. This parameter determines the model that best fits the data.

^4^NC: Not able to calculate due to the initial titer lower than 10^4^ TCID_50_/mL.

^†^Viral titer calculated for 10 mL of analysed sample.

In general, all enveloped viruses tested were inactivated at <3000 J/L and showed non-linear inactivation kinetics, biphasic or Weibull distributions, with low RMSE values resulting in 4D values under 3000 J/L (Figs 1 and 2 and Table 2). The two best non-linear inactivation kinetics with the smallest RMSE are included in Table 2.

Greater ranges of stability were found for non-enveloped viruses. PPV was inactivated at <3000 J/L, but other non-enveloped viruses such as SVA, SVDV and PCV-2 required a greater UV-C dose to be inactivated (Table 2; Fig 3). For example, all three samples of SVDV at 6000 J/L were negative by the microtiter assay. However, the subsequent 3 blind passages performed for each 6000 J/L sample were positive. Afterwards, a titration of 25 mL was done by duplicate, obtaining one positive out of 960 wells, so, it was concluded that there were 0.02 particles/mL (0.014 TCID50%) in the 6000 J/L samples (1 particle/50 mL assayed volume). To further confirm the dose required to inactivate SVDV, three additional blind passages were performed for the three samples irradiated at 9000 J/L, and one was found positive. Titration of the original sample of 9000 J/L (25 mL) was done; one out of 480 inoculated wells was positive. It was calculated that 0.04 particles per milliliter (0.028 TCID50%) were still present in the only positive sample of 9000 J/L (1 particle / 25 mL assayed volume). The most resistant virus was PCV-2, since the total log10 reduction achieved was 2.71 log10 at 9000 J/L.

## Discussion

The efficacy of microbial reduction by UV-C treatment of liquids depends on different factors, including the microorganism used, the opaqueness of the liquid, the presence of suspended particles, and the microbial contamination level at the starting point [46]. Animal plasma from slaughterhouse is an opaque liquid; therefore, UV-C radiation does not penetrate this liquid. This problem has been overcome by introducing turbulent flow achieving that the whole volume of liquid passes close to the UV-C radiation source [25–30]. The UV-C system used in this work can be expanded to efficiently process large volumes of liquid compatible with commercial production of fruit juices, wine [26,38] and milk [47]. It is important to differentiate this unique design of the SurePure Turbulator system for treatment of high volume of opaque liquids from other UV systems developed for treatment of human plasma fractions [25,27] that are designed for treatment of small plasma fraction bags under gentle agitation but cannot be scalable to the volumes used by the commercial SDAP industry.

A preliminary step to implement industrial plasma irradiation with UV-C consisted of demonstrating such treatment does not affect the functionality of proteins present in SDAP. Similar growth and health performance of pigs fed diets with UV-SDAP versus non-irradiated SDAP has been reported [31,48].

The next step was to investigate the inactivation capacity of the system on possible contaminating viruses in the plasma. For this purpose, selected viruses representing enveloped and non-enveloped, DNA and RNA, single-stranded and double-stranded viruses of importance for porcine industry were spiked into SDAP and irradiated. Furthermore, the different viruses tested may be considered surrogates for other untested viruses belonging to the same family or genus, although it is always recommended to test specifically each particular virus [49].

In the present study, UV-C radiation was more effective inactivating tested enveloped viruses compared to the non-enveloped viruses. Enveloped viruses could not be detected when the UV-C dose was >3000 J/L. The best fit lines describing inactivation kinetics of the enveloped viruses were curvilinear biphasic equations. This could be due to lack of more points of collection; in many cases the last data point(s) appeared to be beyond the minimum UV-C radiation required to inactivate the entire virus in the sample, creating an apparent curvilinear shape. Cutler et al, [50] reported biphasic inactivation kinetics of PRRSV, BVDV and SIV using a different UV-C delivery system and with the virus suspended in culture media rather than blood plasma. Although almost a lineal response was observed in the present study, the best model for BVDV was Weibull, and biphasic plus shoulder for SIV. Nevertheless, in both studies, despite using two different irradiation equipment (here versus the used by Cutler et al. [49]), the relative sensitivity of the virus to UV-C inactivation was similar with PRRSV being more sensitive than BVDV or SIV.

A total inactivation (3.16 log) of BVDV was achieved at 3000 J/L. The inactivation kinetics obtained with BVDV is very similar to the one observed in the other tested pestivirus, CSFV, which could indicate that members of the same genus could respond similarly to UV-C treatment.

PRV titer decreased more than 4 log at UV-C irradiation treatment < 3000 J/L, which represented a better inactivation ratio than that reported by another experiment using Riboflavin-UV photochemical based technology [49]. The degree of inactivation by UV-C irradiation may depend on the technology used [51].

To the authors’ knowledge, this is the first study showing effective results in the inactivation of PEDV by means of UV-C.

The non-enveloped viruses that were tested appeared to be more resistant to inactivation by UV-C radiation. The inactivation curve for PPV was consistent with previously published data [31], with apparent linear inactivation in response to increasing UV-C dose. Likewise, the inactivation curve for SVA appeared to be linear except for the highest UV-C dose. The inactivation curve for SVDV showed minimal inactivation at low UV-C dose, suggesting that a minimal UV-C dose was necessary before virus was inactivated. This has been reported by others [40] who suggested that for some virus inactivation does not occur until a minimum UV-C dose is achieved. However, in this experiment appears to be another shoulder in the inactivation curve at intermediate UV-C dosage (1500 and 3000 J/L). Furthermore, while the highest doses appeared to inactivate all the virus, subsequent passages detected very low residual viral infectivity. The inactivation curve for PCV-2 demonstrated that UV-C radiation inactivated this virus; however, even at the highest dose (9000 J/L), residual levels of PCV-2 were recovered. The presence of a resistant viral subpopulation has been attributed to the ability of some viruses to take advantage of host cell repair mechanisms [52], or in some cases they can code for their own repair machinery [53]. It has also been argued that viral particle clumping, particularly with other cells or debris, may shield some virus from UV-C radiation [50].

In the present study, a difference between the theoretical titer in the spiked plasma and the measured titer at time zero was observed. The difference was greater with enveloped viruses than with non-enveloped viruses. Generally, enveloped viruses are more sensitive to environmental conditions such as salts, temperature changes, freezing and thawing or presence of anticoagulants [19] and could explain the reduction in titer before irradiation compared to the theoretical titer [4].

There was not a clear relationship between rate of inactivation and genome type or size. Theoretically, DNA is more sensitive to UV-C due to the presence of thymine [54]. In contrast, DNA repair can reduce the UV-C effect, especially for dsDNA viruses [55]. In the present study, PRV (a dsDNA virus) had 4D reduction estimated at 1612 J/L, which was not apparently different from the 4D reduction values obtained for other tested RNA viruses (PEDV and SIV). Obtained results with viruses evaluated in this study suggest that UV-C inactivation susceptibility of both types of viral genome was similar.

It has been suggested that the longer the genome, the higher the susceptibility to UV-C damage [56]. However, based on the present results, increased genome size did not appear to increase virus susceptibility to UV-C radiation when spiked into commercially collected animal plasma and exposed to UV-C. Only a weak linear trend (r^2^ = 0.43) was found in the current data when comparing genome size to the 4D of viruses (without outlier’s results PEDV and PRV). Curiously, Wang et al. [25] found that viruses with large genomes (*Adenovirus* and *Reovirus*) were inactivated with higher UV-C doses than viruses with shorter genomes. These authors indicated that their unexpected survival curves compared with other UV-C irradiation studies was difficult to explain, and suggested differences in the shape, size and lamp geometry of UV-irradiation systems as well as in protein concentrations and composition of process streams to explain their results. Also, genome configuration may affect the virus sensitivity to UV-C radiation. For example, both PPV and PCV-2 have a relatively small genome size (5.07 and 1.77 Kb, respectively). However, estimated 4D for PPV was 2161 J/L while that for PCV-2 was >9000 J/L. It is possible that the linear configuration of PPV genome could be more easily damaged, whereas the circular genome of PCV-2 may provide resistance to the formation of thymidine dimers by UV-C radiation. In any case, the present study should be considered exploratory in nature regarding the relationship between size and type of the genome and susceptibility to inactivation by UV-C irradiation.

In addition to the photochemical damage of UV-C irradiation on nucleic acids, UV-C also induces reactive oxygen species that may interact with the external lipid bilayer membrane of enveloped viruses [57], causing lipid peroxidation [58]. This effect may further explain why enveloped viruses were very susceptible to UV-C irradiation. In fact, in the present study, irradiation doses used were between 30–375 times higher than those described in the literature [59]. These differences could be due to the presence of proteins and other molecules in solution, to the possibility of virus clumping with these molecules, to the greater opacity of the liquid against UV, to the higher basal absorbance and/or to the darker color of the liquid plasma solution compared to other more transparent solution like water or other buffers like PBS. Nevertheless, our results are closer to irradiation values found in other opaque raw materials like milk or juices [26,60].

Overall, the SurePure UV-C Turbulator design was effective in inactivating a wide variety of virus spiked into commercially collected liquid animal plasma. Data from the present study indicated that the UV-C irradiation of liquid plasma is a technology that could provide an additional inactivation step to the industrial production process for SDAP. Exposure to UV-C is extensively used for the disinfection of liquid media and surfaces due to its germicidal activity [23,24]. Therefore, UV-C irradiation of liquid plasma during the manufacturing process for SDAP has a potential application for inactivation of microbial contaminants without causing negative effects on the nutritional or physical qualities of the treated material [51,61]. Furthermore, the UV-C mechanism of inactivation (DNA or RNA damage) is different than the thermal inactivation provided by the spray-drying step. In consequence, the UV-C step can be considered independent from the spray-drying process and may be a synergistic process as both procedures have different inactivation targets.

In conclusion, obtained results demonstrate UV-C as a suitable technology to be applied in the manufacturing process of SDAP as a redundant biosafety step for the inactivation of viruses of concern for the livestock industry.

## Supporting information

S1 Table PRV, PRRSV and PEDV titration results for each triplicate at each time/dose. (PDF)

S2 Table BVDV, CSFV and SIV titration results for each triplicate at each time/dose. (PDF)

S3 Table PPV, SVDV and PCV-2 titration results for each triplicate at each time/dose. (PDF)

S4 Table SVA titration results for each triplicate at each time/dose. (PDF)
