# Supplementary material for: Evaluation of the effectiveness of the SurePure Turbulator ultraviolet-C irradiation equipment on inactivation of different enveloped and non-enveloped viruses inoculated in commercially collected liquid animal plasma
Source: PLoS One. 2019 Feb 21;14(2):e0212332. doi: 10.1371/journal.pone.0212332 (PMC6383881; doi:10.1371/journal.pone.0212332)
Supplement: S1 Table — (PDF) [file pone.0212332.s001.pdf]

S1 Table 1. PRV, PRRSV and PEDV titration results for each triplicate at each time/dose. Dose was calculated as a UV-fluence received per unit of time. These data were used for GInaFiT analysis.

PRV		
DOSE (J/L)	TIME (min)	Log10/mL
0	0.00	4.53
0	0.00	4.53
0	0.00	4.53
750	3.52	3.46
750	3.52	3.95
750	3.52	3.94
1500	7.39	1.37
1500	7.39	1.35
1500	7.39	1.35
3000	15.06	-1.69
3000	15.06	-1.69
3000	15.06	-1.69
6000	29.48	-1.69
6000	29.48	-1.69
6000	29.48	-1.69
9000	44.07	-1.69
9000	44.07	-1.69
9000	44.07	-1.69

PRRSV		
DOSE (J/L)	TIME (min)	Log10/mL
0	0.00	4.07
0	0.00	3.89
0	0.00	4.06
750	3.52	1.47
750	3.52	1.03
750	3.52	1.50
1500	7.39	-1.69
1500	7.39	-1.69
1500	7.39	-1.69
3000	15.06	-1.69
3000	15.06	-1.69
3000	15.06	-1.69
6000	29.48	-1.69
6000	29.48	-1.69
6000	29.48	-1.69
9000	44.07	-1.69
9000	44.07	-1.69
9000	44.07	-1.69

PEDV		
DOSE (J/L)	TIME (min)	Log10/mL
0	0.00	4.01
0	0.00	4.01
0	0.00	4.20
750	3.54	2.30
750	3.54	2.17
750	3.54	1.70
1500	7.45	1.12
1500	7.45	1.24
1500	7.45	1.22
3000	15.24	-1.69
3000	15.24	-1.69
3000	15.24	-1.69
6000	30.35	-1.69
6000	30.35	-1.69
6000	30.35	-1.69
9000	45.35	-1.69
9000	45.35	-1.69
9000	45.35	-1.69
