# Supplementary material for: Evaluation of the effectiveness of the SurePure Turbulator ultraviolet-C irradiation equipment on inactivation of different enveloped and non-enveloped viruses inoculated in commercially collected liquid animal plasma
Source: PLoS One. 2019 Feb 21;14(2):e0212332. doi: 10.1371/journal.pone.0212332 (PMC6383881; doi:10.1371/journal.pone.0212332)
Supplement: S2 Table — (PDF) [file pone.0212332.s002.pdf]

S2 Table 2. BVDV, CSFV and SIV titration results for each triplicate at each time/dose. Dose was calculated as a UV-fluence received per unit of time. These data were used for GInaFiT analysis.

BVDV		
DOSE (J/L)	TIME (min)	Log10/mL
0	0.00	3.94
0	0.00	4.12
0	0.00	4.43
750	3.51	3.25
750	3.51	3.50
750	3.51	3.35
1500	7.34	1.57
1500	7.34	1.61
1500	7.34	1.72
3000	14.52	-2.69
3000	14.52	-2.69
3000	14.52	-2.69
6000	29.11	-2.69
6000	29.11	-2.69
6000	29.11	-2.69
9000	43.04	-2.69
9000	43.04	-2.69
9000	43.04	-2.69

CSFV		
DOSE (J/L)	TIME (min)	Log10/mL
0	0.00	4.08
0	0.00	4.12
0	0.00	4.08
750	3.52	3.38
750	3.52	3.16
750	3.52	3.16
1500	7.39	0.64
1500	7.39	0.61
1500	7.39	0.66
3000	15.06	-1.69
3000	15.06	-1.69
3000	15.06	-1.69
6000	29.48	-1.69
6000	29.48	-1.69
6000	29.48	-1.69
9000	44.07	-1.69
9000	44.07	-1.69
9000	44.07	-1.69

SIV		
DOSE (J/L)	TIME (min)	Log10/mL
0	0.00	5.05
0	0.00	5.14
0	0.00	5.08
750	3.53	4.01
750	3.53	4.06
750	3.53	4.11
1500	7.37	1.61
1500	7.37	1.63
1500	7.37	1.72
3000	14.53	-1.69
3000	14.53	-1.69
3000	14.53	-1.69
6000	29.06	-1.69
6000	29.06	-1.69
6000	29.06	-1.69
9000	42.39	-1.69
9000	42.39	-1.69
9000	42.39	-1.69
