# Supplementary material for: Evaluation of the effectiveness of the SurePure Turbulator ultraviolet-C irradiation equipment on inactivation of different enveloped and non-enveloped viruses inoculated in commercially collected liquid animal plasma
Source: PLoS One. 2019 Feb 21;14(2):e0212332. doi: 10.1371/journal.pone.0212332 (PMC6383881; doi:10.1371/journal.pone.0212332)
Supplement: S3 Table — (PDF) [file pone.0212332.s003.pdf]

S3 Table 3. PPV, SVDV and PCV-2 titration results for each triplicate at each time/dose. Dose was calculated as a UV-fluence received per unit of time. These data were used for GInaFiT analysis.

PPV		
DOSE (J/L)	TIME (min)	Log10/mL
0.00	0.00	4.02
0.00	0.00	3.93
0.00	0.00	4.05
750.00	3.52	3.37
750.00	3.52	3.16
750.00	3.52	3.16
1500.00	7.39	1.70
1500.00	7.39	1.65
1500.00	7.39	1.72
3000.00	15.06	-1.69
3000.00	15.06	-1.69
3000.00	15.06	-1.69
6000.00	29.48	-1.69
6000.00	29.48	-1.69
6000.00	29.48	-1.69
9000.00	44.07	-1.69
9000.00	44.07	-1.69
9000.00	44.07	-1.69

SVDV		
DOSE (J/L)	TIME (min)	Log10/mL
0	0.00	4.08
0	0.00	4.08
0	0.00	4.08
750	3.52	4.10
750	3.52	4.03
750	3.52	4.11
1500	7.39	1.70
1500	7.39	1.65
1500	7.39	1.72
3000	15.06	1.48
3000	15.06	1.46
3000	15.06	1.46
6000	29.48	-1.69
6000	29.48	-1.69
6000	29.48	-1.69
9000	44.07	-1.39
9000	44.07	-1.39
9000	44.07	-1.39

PCV-2		
DOSE (J/L)	TIME (min)	Log10/mL
0	0.00	3.32
0	0.00	3.12
0	0.00	3.33
750	3.51	1.67
750	3.51	1.64
750	3.51	1.73
1500	7.34	1.58
1500	7.34	1.49
1500	7.34	1.55
3000	14.52	1.49
3000	14.52	1.47
3000	14.52	1.49
6000	29.11	0.78
6000	29.11	0.72
6000	29.11	0.69
9000	43.04	0.56
9000	43.04	0.55
9000	43.04	0.53
