# Supplementary material for: Evaluation of the effectiveness of the SurePure Turbulator ultraviolet-C irradiation equipment on inactivation of different enveloped and non-enveloped viruses inoculated in commercially collected liquid animal plasma
Source: PLoS One. 2019 Feb 21;14(2):e0212332. doi: 10.1371/journal.pone.0212332 (PMC6383881; doi:10.1371/journal.pone.0212332)
Supplement: S4 Table — (PDF) [file pone.0212332.s004.pdf]

S4 Table 4. SVA titration results for each triplicate at each time/dose. Dose was calculated as a UV-fluence received per unit of time. These data were used for GInaFiT analysis.

SVA		
**DOSE (J/L)**	**TIME (min)**	**Log10/mL**
0	0.00	4.68
0	0.00	4.50
0	0.00	4.86
750	4.01	4.15
750	4.01	4.05
750	4.01	4.15
1500	7.52	3.02
1500	7.52	2.92
1500	7.52	3.02
3000	15.25	0.93
3000	15.25	1.05
3000	15.25	0.85
6000	30.09	-1.69
6000	30.09	-1.69
6000	30.09	-1.69
9000	44.14	-1.69
9000	44.14	-1.69
9000	44.14	-1.69
